# Bactericidal Activity of Usnic Acid-Chitosan Nanoparticles against Persister Cells of Biofilm-Forming Pathogenic Bacteria

**DOI:** 10.3390/md18050270

**Published:** 2020-05-20

**Authors:** Fazlurrahman Khan, Hongsik Yu, Young-Mog Kim

**Affiliations:** 1 Institute of Food Science, Pukyong National University, Busan 48513, Korea; fkhan055@pknu.ac.kr; 2 Food Safety and Processing Research Division, National Institute of Fisheries Science, Busan 46083, Korea; yhspknu@korea.kr; 3 Department of Food Science and Technology, Pukyong National University, Busan 48513, Korea

**Keywords:** anti-persister, bacteria, chitosan, nanoparticles, pathogens, usnic acid, UA-CS NPs

## Abstract

The present study aimed to prepare usnic acid (UA)-loaded chitosan (CS) nanoparticles (UA-CS NPs) and evaluate its antibacterial activity against biofilm-forming pathogenic bacteria. UA-CS NPs were prepared through simple ionic gelification of UA with CS, and further characterized using Fourier transform infrared spectroscopy, X-ray diffraction, and field-emission transmission electron microscopy. The UA-CS NPs presented a loading capacity (LC) of 5.2%, encapsulation efficiency (EE) of 24%, and a spherical shape and rough surface. The maximum release of UA was higher in pH 1.2 buffer solution as compared to that in pH 6.8 and 7.4 buffer solution. The average size and zeta potential of the UA-CS NPs was 311.5 ± 49.9 nm in diameter and +27.3 ± 0.8 mV, respectively. The newly prepared UA-CS NPs exhibited antibacterial activity against persister cells obtained from the stationary phase in batch culture, mature biofilms, and antibiotic-induced gram-positive and gram-negative pathogenic bacteria. Exposure of sub-inhibitory concentrations of UA-CS NPs to the bacterial cells resulted in a change in morphology. The present study suggests an alternative method for the application of UA into nanoparticles. Furthermore, the anti-persister activity of UA-CS NPs may be another possible strategy for the treatment of infections caused by biofilm-forming pathogenic bacteria.

## 1. Introduction

Studies have shown that the formation of persister cells by most pathogenic bacteria is a challenging task when treating infections using antimicrobial agents [1,2,3]. Persisters are a small population of metabolically inactive (dormant) cells that exhibit tolerance against antibiotics and several environmental stresses [3,4]. Persister cells are heterogeneously present in the planktonic stationary population as well as in the biofilm state [5,6]. Reports have shown that the formation of persister cells occurs stochastically as well as under several environmental factors, such as nutrient deficiency, oxidative stress, DNA damage, drop in cellular ATP, and sub-inhibitory concentration of antibiotics [1,3,7]. Apart from these factors, the biofilm environment provides additional protection against the entry of the antibiotic to the persister cells [8], which are encased beneath the biofilm matrix composed of extracellular polymeric substances (EPS) such as extracellular DNA, exopolysaccharide, and proteins [9]. Once the aforementioned environmental stress releases/drops, this subpopulation begins to multiply and recurrent infection occurs. The tolerance properties of persister cells lead to the identification of several compounds either from natural sources or by chemical synthesis [4]. Although several achievements have been made in the eradication of the persister cells as summarized by Defraine et al. [4], due to the varied nature and diversity in their occurrence, there is still the need to explore more anti-persister agents.

The naturally isolated antimicrobial agents are considered eco-friendly, cost-effective in isolation, biocompatible, and with the least cytotoxic effect [10,11,12]. Recent trends have shown that the bioactive agents obtained from marine organisms receive significant attention due to their outstanding biological, chemical, and physical properties [10,11]. Usnic acid (UA) is a secondary metabolite isolated from lichen and has received great attention due to its diverse antimicrobial [13,14,15], anticancer [16], and wound healing properties [17]. However, poor water solubility limits its application [13,18]. It has also been used for the control of biofilm-forming pathogens [13,19]. With the advance of nanotechnology, the application of nanoformulations or combinations of different bioactive agents has led to the easy treatment of infections associated with pathogenic bacteria due to the easy delivery system, high efficiency, and minimization of drug toxicity [20]. In particular, the application of polymeric nanoparticles for the encapsulation or loading of drugs has received more attention, since it enhances stability and controlled release, improves solubility, protects against environmental oxidation, targets delivery, and reduces the toxic effect of the drugs [21,22,23,24].

Chitosan is a natural linear polycationic amino polysaccharide derived from chitin by enzymatic and chemical methods [10,25]. Due to its biocompatibility, nontoxicity, biodegradability, and cost-effectiveness in isolation, it has been used for different purposes in biomedical, food, and pharmaceutical industries [10,26,27]. In addition, it has been widely applied for the encapsulation or loading of bioactive compounds via different preparation methods [22,28,29,30]. Hence, the present study aimed to encapsulate UA into chitosan nanoparticles (CS NPs) through an ionotropic gelification method [28]. The newly prepared UA-CS NPs exhibited bactericidal activity towards the persister cells of gram-positive (e.g., *Listeria monocytogenes* and *Staphylococcus aureus*) and gram-negative bacteria (*Escherichia coli* and *Pseudomonas aeruginosa*), which are produced in stationary phase in a batch culture, in the biofilm state, or antibiotic-induced. 

## 2. Result and Discussion

### 2.1. Preparation and Characterization of UA-CS NPs

**Figure 1:**
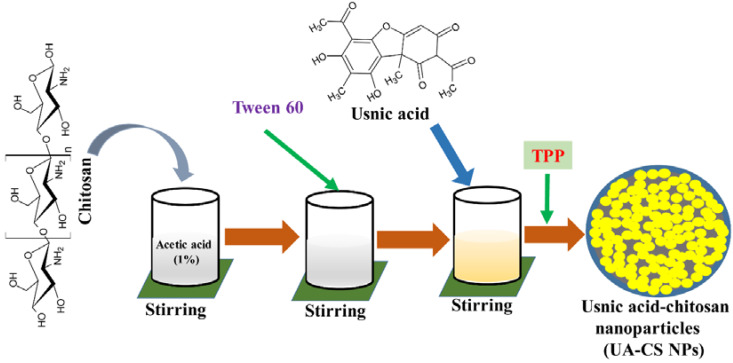
Diagrammatic representation of preparation of the usnic acid-loaded chitosan nanoparticles (UA-CS NPs).

Biocompatibility, easy availability, cost-effectiveness, and biodegradability of chitosan make it widely accepted as a carrier of several hydrophobic, hydrophilic, and unstable drugs [10,26]. In the present study, we selected low-molecular-weight chitosan for the encapsulation of UA through a simple ionotropic gelification method using tripolyphosphate (TPP). A schematic representation of the preparation of UA-CS NPs is shown in Figure 1. Due to the yellow color of the UA, the final UA-CS NPs also appears light yellowish in color. 

The loading of UA into CS NPs was confirmed by release assay (using UV-Vis spectrophotometry) and FTIR analysis. Reports have shown that UA presents various absorption spectra when dissolved in different solvents [31,32,33]. In the aqueous solvent, two maximum absorption peaks were observed at 231 nm and 287 nm [31]. Dasgupta et al. [19] reported that UA has one sharp maximum absorption peak at 244 nm, a broad maximum absorption peak at 278 nm, and a prominent shoulder at 337 nm and they quantitatively determined the release of UA by measuring the absorption at 337 nm.

**Figure 2:**
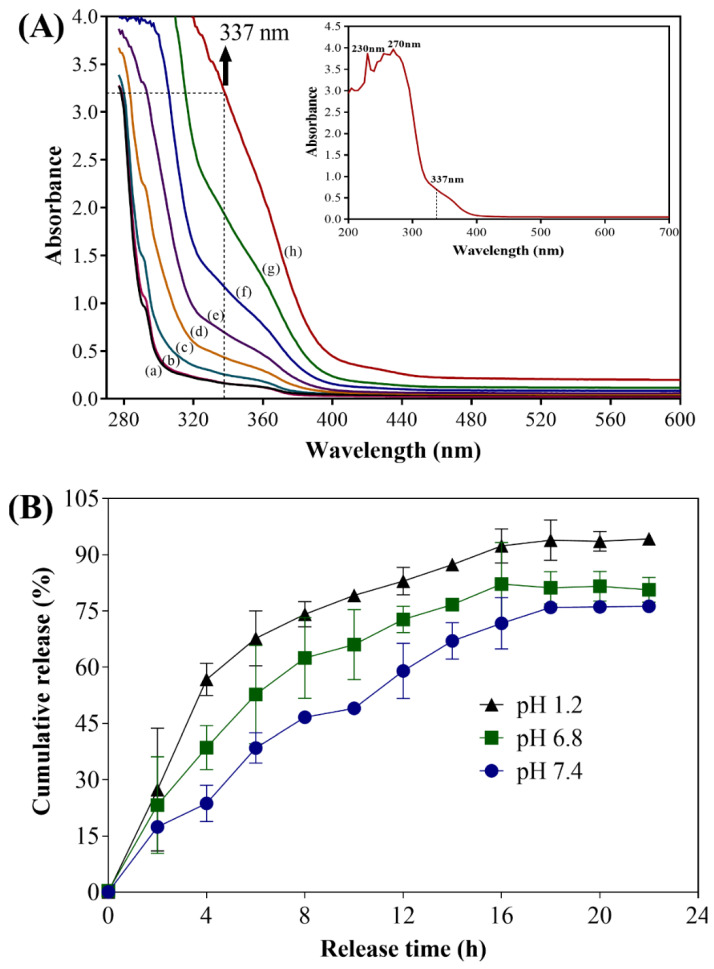
(**A**) Time-dependent UV-Vis absorption spectrum of UA released in phosphate-buffered saline (PBS) (pH 7.4) from CS NPs. (a) CS NPs at 22 h, (b) UA-CS NPs at 0 h, (c) UA-CS NPs at 2 h, (d) UA-CS NPs at 4 h, (e) UA-CS NPs at 8 h, (f) UA-CS NPs at 10 h, (g) UA-CS NPs at 22 h, and (h) UA standard (512 µg/mL). The inset is UV-Vis absorption spectra of UA showing two maximum absorption peaks (230 and 270 nm) and prominent shoulder at 337 nm. (**B**) Cumulative release of UA from CS NPs, which was prepared from 1.2% chitosan and 0.5% UA in the presence of 0.5% tripolyphosphate (TPP). The release of UA from CS NPs was conducted in PBS at pH 1.2, 6.8, and 7.4 and the amount of UA released was quantified by measuring the absorbance at 337 nm.

Hence, to check the encapsulation of UA in the CS NPs, we determined the release of UA in the buffered solution by measuring the absorption at 337 nm [19]. The release of UA from CS NPs was conducted in phosphate-buffered saline (PBS) at pH 1.2, 6.8, and 7.4 and the amount of UA released was determined at different time intervals. The supernatant collected at different time intervals from the release assays showed an increasing absorption spectrum of UA at 337 nm (Figure 2A). Figure 2A shows the time-dependent UV-Vis absorption spectrum of UA released from the CS NPs. The UV-vis absorption spectra of UA showing characteristic maximum absorption peaks (230 and 270 nm) along with a prominent shoulder at 337 nm in PBS (pH 7.4) are separately presented in the inset of Figure 2A. The results clearly showed an increase in the absorbance at 337 nm, indicating the release of UA from CS NPs. The supernatant obtained from the control solution (containing only CS NPs) did not show any characteristic absorption peaks (Figure 2A, a).

Controlled release of UA was observed at pH 1.2, 6.8, and 7.4 (Figure 2B). The initial fast release of UA (pH 7.4) was 49 ± 1.3% in 10 h, whereas the maximum release of 76.2 ± 0.9% occurred at 22 h of incubation. The release of UA at pH 6.8 was slightly higher (80.6 ± 1.9% at 22 h of incubation) compared to that at pH 7.4 (Figure 2B). Furthermore, the release of UA in acidic conditions (pH 1.2) was found to be 94.2 ± 0.9% at 22 h of incubation, which is higher among all the conditions (Figure 2B). A similar profile of drug release at different pH has been reported previously [21,34]. Reports have shown that the release of drugs is greatly affected by the pH of the media, and a high release of drug in acidic media is due to the swelling and dissolution of CS NPs [21,35]. Several mechanisms have been proposed to explain the release of the drug encapsulated in the CS NPs, such as diffusion, degradation of polymeric materials, and erosion [36,37]. A recent study showed that UA loaded onto a bioadhesive film prepared from hydrogel released up to 87% UA during the 24 h incubation [38].

The controlled release of several drugs from CS NPs has also been reported for other drugs, such as chlorogenic acid and carvacrol [21,39]. The loading efficiency (LC) of UA in CS NPs was 5.2% in the presence of 0.5% TTP. Similarly, the encapsulation efficiency (EE) of UA in CS NPs was 24.0%. Several naturally derived active molecules have been successfully encapsulated into CS NPs [22,34,39,40]. The percentages of EE and LC found in the present study were in close agreement with the previously reported encapsulation for other naturally derived molecules using the same procedure for the preparation of the nanoparticles [22]. The LC or EE of biologically derived material into chitosan was lower due to the higher cross-linkage, which might cause shrinkage and squeezing out the drug [22,41]. Previous reports have shown that EE decreased in the presence of high concentrations of TPP (1.0%) for the loading of eugenol or epigallocatechin gallate to the CS NPs [22,42]. 

**Figure 3:**
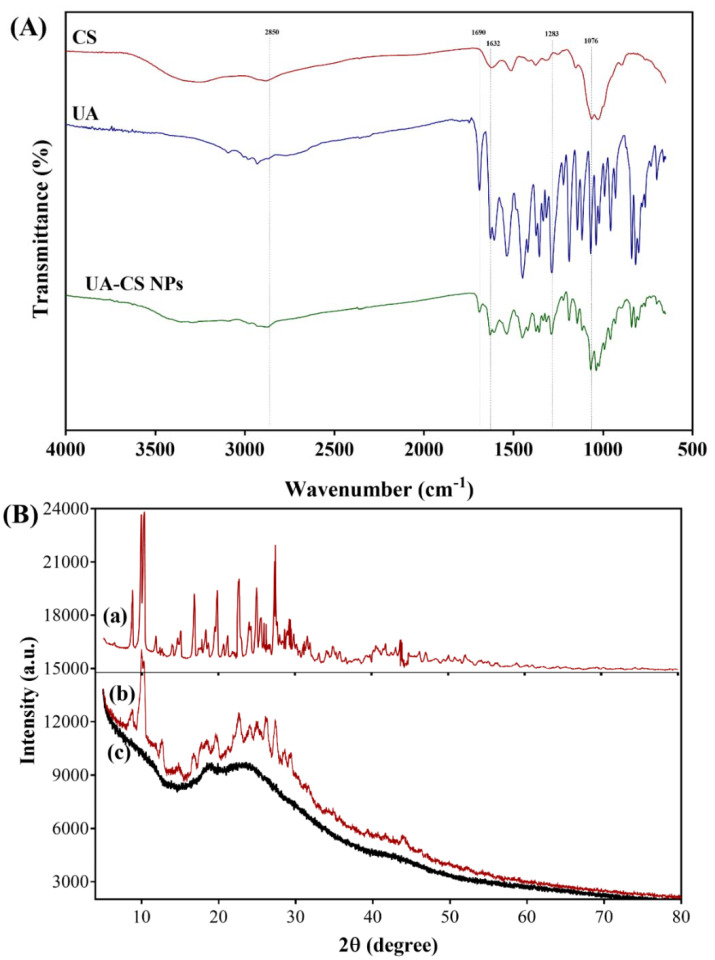
(**A**) FTIR spectra of UA-CS NPs prepared from 1.2% chitosan and 0.5% UA in the presence of 0.5% TPP and (**B**) XRD-spectra of UA (a), UA-CS NPs (b) and CS NPs (c).

The physical interaction between the components in the UA-CS NPs was determined through FTIR. The FTIR spectra of UA corresponds to several characteristic peaks in the IR spectrum, as reported earlier [19,38,43]. A unique characteristic peak in the FTIR spectrum of UA at 1690 cm^−1^ was observed, which was previously identified as the –C=O stretching of conjugated cyclic ketones. Similarly, the peak at 1632 cm^−1^ corresponds to the methyl ketone. The bands at 1283 and 1072 cm^−1^ correspond to the antisymmetric and symmetric m(COC) aryl alkyl ether modes, as identified by Pagano et al. [38]. The chitosan also showed some characteristic peaks in the FTIR spectrum, including 3356 (-OH and –NH_2_ stretching), 2875 (-CH stretching), 1647 (C=O stretching of amide I), 1153 (asymmetric stretching of C-O-C bridge), and 1025 cm^−1^ (C-O stretching) (Figure 3A). These characteristic peaks appeared in chitosan, as reported in several studies [44,45]. 

Along with the appearance of UA peaks, some characteristic peaks of chitosan have also been observed in the FTIR spectra of UA-CS NPs, which indicates that there might be some physical interaction between UA and CS. Thus, based on the release assay, and FTIR results, we concluded that the UA was encapsulated in the chitosan polymeric nanoparticles. Furthermore, the crystallographic structure of the UA-CS NPs was determined using XRD. The XRD spectra showed several sharp characteristics as well as diffuse peaks in the diffraction pattern of the UA-CS NPs (Figure 3B). The characteristics peaks of UA were observed at different 2ϴ values such as 10.3°, 14.7°, 18.5°, 22.7°, 24.9°, and 27.4° (Figure 3B, a). Similar peaks in UA has also been reported previously [46,47], which are also present in the UA-CS NPs (Figure 3B, b). In case of CS NPs, an intense diffraction peak at 2 ϴ = 22.6° has been observed (Figure 3B, c) which is also present in the UA-CS NPs spectra along with some characteristic peaks of UA. 

**Figure 4:**
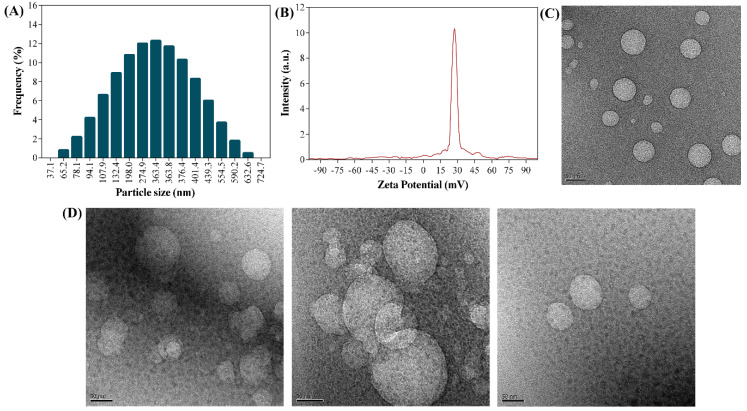
(**A**) Dynamic light scattering (DLS) particle size distribution of UA-CS NPs prepared from 1.2% chitosan and 0.5% UA in the presence of 0.5% TPP, (**B**) Zeta potential of UA-CS NPs prepared from 1.2% chitosan and 0.5% UA in the presence of 0.5% TPP, (**C**) FE-TEM micrograph of CS NPs prepared from 1.2% chitosan in the presence of 0.5% TPP, and (**D**) FE-TEM micrograph of UA-CS NPs prepared from 1.2% chitosan and 0.5% UA in the presence of 0.5% TPP.

The diameter (hydrodynamic) of the UA-CS NPs was determined using dynamic light scattering (DLS) (Figure 4A). The particle size of the UA-CS NPs in the presence of 0.5% TPP had an average diameter of 311.5 ± 49.9 nm (86.8% of the particles have the dimension in the range of 107.9 to 439.3 nm) and a polydispersity index (PDI) value of 0.38. Similarly, the average size and PDI value of UA-CS NPs in the presence of 1.0% TPP was 401.7 ± 66.9 nm in diameter (87.5% of the particles have the dimension in the range of 150.2 to 698.2 nm) and 0.41, respectively. The particle size and PDI of CS NPs in the presence of 0.5% TPP were 235.2 ± 47.3 nm (87.5% of the particles have the dimension in the range of 82.2 to 421 nm) and 0.60, respectively, whereas in the presence of 1.0% TPP were 286.9 ± 52.3 nm (85.8% of the particles have the dimension in the range of 163.2 to 332.7 nm) and 0.49, respectively. The results showed that by increasing the concentration of TPP, the size of nanoparticles (loaded or unloaded with UA) was found to be increased, although, UA loaded CS NP showed larger size as compared to the unloaded CS NPs. The formation of large-sized nanoparticles have been reported before as a result of (1) high degree of cross-linking between chitosan and TPP in the presence of high concentration of TPP [22,41], (2) loading of drugs [21,22,41,48], and (3) swelling or aggregation of NPs when dispersed in the aqueous medium [22,49]. The molecular weight, degree of deacetylation, and concentration of chitosan are other factors that have been reported to affect the size of CS NPs [50]. Our results are in close agreement with previously reported results for chitosan-loaded drug nanoparticles [22,48]. The zeta potential of the UA-CS NPs prepared from 1.2% CS and 0.5% UA in the presence of 0.5% TPP was +27.3 ± 0.8 mV (Figure 4B), whereas the zeta potential for CS NPs prepared in the presence of 0.5% TPP was +35.6± 1.9 mV, suggesting that the decrease in zeta potential might be due to the loading of UA. Similar effects have been observed in several studies where the loading of drugs decreases the zeta potential [21,22]. The morphology of CS NPs, as determined by FE-TEM, was found to be spherical (Figure 4C). Similarly, the morphology of the UA-CS NPs was also spherical and had a rough surface (Figure 4D). Several reports showed CS NPs loaded with drugs are spherical in shape [21,22,48]. 

### 2.2. Minimum Inhibitory Concentration (MIC) Values of UA-CS NPs against Gram-Positive and Gram-Negative Bacteria

**Figure 5:**
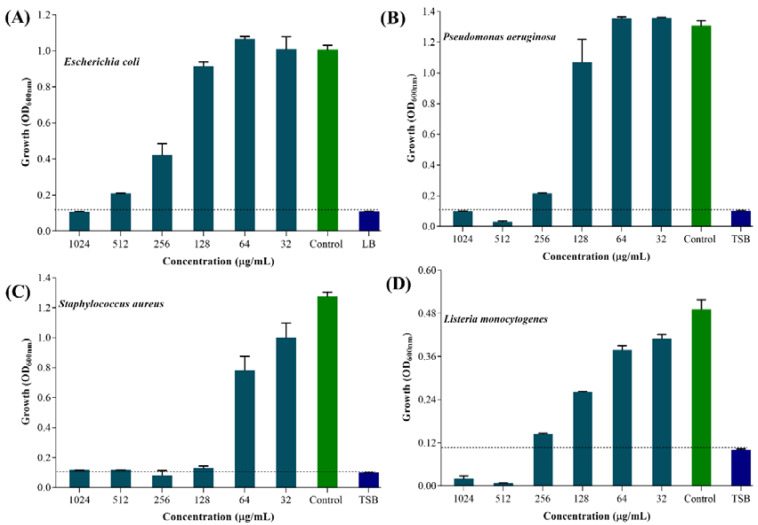
MIC determination of UA-CS NPs against gram-positive and gram-negative bacteria. (**A**) Bactericidal effect of UA-CS NPs against *E. coli*, (**B**) Bactericidal effect of UA-CS NPs against *P. aeruginosa*, (**C**) Bactericidal effect of UA-CS NPs against *S. aureus* and (**D**) Bactericidal effect of UA-CS NPs against *L. monocytogenes*. The MIC was determined by microbroth dilution in a 96-well microtiter plate at 37 ℃ for 24 h under shaking conditions. The cell growth was determined by measuring optical density (OD) at 600 nm. The positive cell growth was considered when the OD_600_ value was found to be > 0.1, since the OD_600_ of broth was found to be ~ 0.1. Each bar is represented as the means of three replicates ± standard deviation.

The MIC value of UA-CS NPs against each selected pathogenic bacterium was determined by the microbroth dilution method in a 96-well microplate. The results showed different MIC values of UA-CS NPs against the tested bacteria (Figure 5). The absorbance of the blank (broth medium) was approximately 0.10; hence, an absorbance of the test sample > 0.10 was considered as positive cell growth. The MIC value of UA-CS NPs against *E. coli* was 1024 µg/mL (Figure 5A), whereas the MIC value against *P. aeruginosa* and *L. monocytogenes* was 512 µg/mL (Figure 5B,D). The MIC value against *S. aureus* was 256 µg/mL (Figure 5C). The antibacterial activity of UA against some of the pathogenic bacteria, either in the free form or loaded on the polymeric material, has been reported [13,19,51,52,53].

The free form of UA exhibited antibacterial activity at 150–200 µg/mL (*Streptococcus pyogenes*) [51], 64 µg/mL (*S. aureus*) [52], and 32 μg/mL (*Mycobacterium aureum* and *M. tuberculosis*) [54]. Similarly, the concentration of the loaded form (a complex of discrete combination of UA and polyacrylamide) of UA exhibiting antibacterial activity against *S. epidermidis* was 5–10 µg/mL [53]. Francolini et al. [13] reported a MIC value of UA 64 µg/mL against *S. aureus* and 256 µg/mL against *P. aeruginosa* strain pMF230. The MIC value of UA against *E. coli* is not available; however, a MIC value of 0.39 µg/mL against *L. monocytogenes* was reported using a disc diffusion method [15]. In the present study, the MIC value of UA-CS NPs against all tested strains was one fold higher in the case of *P. aeruginosa* and *S. aureus* compared to the previously reported MIC values for free UA. The higher MIC value of UA-CS-NPs against these bacterial strains might be due to the encapsulation of UA inside the CS NPs, which results in the lowest availability of free UA, since approximately 76% release of UA at pH 7.4 was determined in the release assay (Figure 2B). The maximum amount of UA released at 22 h was approximately 152 ± 1.0 µg/mL, which is below the safety levels of UA, as reported earlier the release of UA from a biodegradable polyester (158 ± 9 µg/mL in 7 days of incubation) [19]. Hence, it can be safe for the application in the biomedical field.

### 2.3. Eradication Effects of UA-CS NPs Towards Different Types of Persister Cells

#### 2.3.1. Anti-Persister Activity of UA-CS NPs Towards Stationary-Phase Cultures

**Figure 6:**
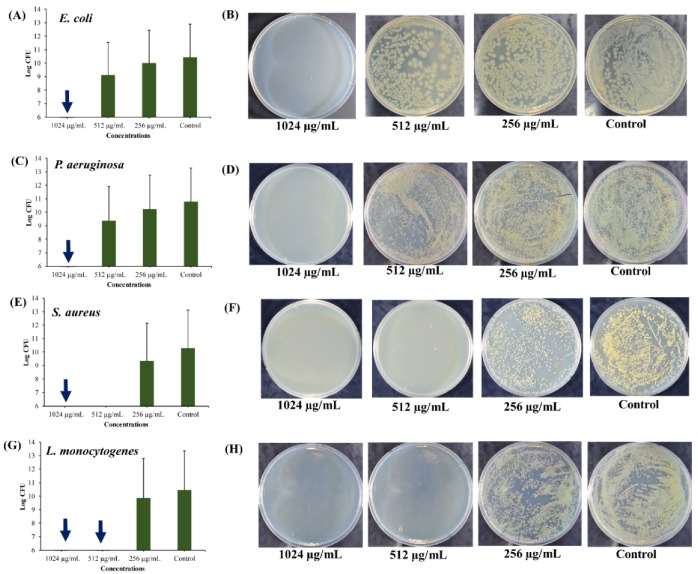
Killing of stationary phase cells by different concentrations of UA-CS NPs. (**A**) Log CFU (colony-forming unit) graph of *E. coli*, (**B**) *E. coli* colonies on TSA (tryptic soy agar) plate, (**C**) Log CFU graph of *P. aeruginosa*, (**D**) *P. aeruginosa* colonies on TSA plate, (**E**) Log CFU graph of *S. aureus*, (**F**) *S. aureus* colonies on TSA plate, (**G**) Log CFU graph of *L. monocytogenes*, and (**H**) *L. monocytogenes* colonies on TSA plate. After incubation, the UA-CS NPs treated cell culture were diluted up to 10^−6^ dilution and 100 µL mixture spread was plated on the TSA plate. Each plate shows a representative image of bacterial colonies. The arrow in the graphs indicate the complete killing of persister cells. Each bar is represented as the means of three replicates ± standard deviation.

As per definition, persister cells are the small subpopulation of the metabolically inactive dormant cells that tolerate the antimicrobial treatment and withstand several other environmental stress conditions [55,56]. These persister cells revert into active cells once the antibiotic exposure drops or any other environmental stress condition is eliminated. Hence, the formation of persister cells has been reported to have some clinical relevance, such as causing chronic or recurrent infections and acting as a reservoir for the emergence of antibiotic-resistant mutants [57,58]. It has been reported that the formation of persister cells is correlated with the growth phase of the bacterial population [6]. Stationary phase cultures of *E. coli*, *P. aeruginosa,* and *S. aureus* present high numbers of persister cells [59]. Hence, it is very important to inactivate the antibiotic-tolerant persister cells produced in the stationary phase of batch cultures of pathogenic bacteria. The killing of the stationary phase-derived persister cells depended on the concentration of UA-CS NPs (Figure 6). The complete killing of persister cells of *E. coli* and *P. aeruginosa* occurred at 1024 µg/mL of UA-CS NPs (Figure 6A,C). However, the complete killing of persister cells of *S. aureus* occurred at 1024 µg/mL (Figure 6E); whereas, in *L. monocytogenes* it occurred at 1024 and 512 µg/mL of UA-CS NPs (Figure 6G). The representative agar plate with bacterial colonies obtained from the cell culture treated with different concentrations of UA-CS NPs is shown in Figure 6B,D,F,H. From the aforementioned results, we concluded that UA-CS NPs completely kill the persister cells of *E. coli* and *L. monocytogenes* at MIC values, whereas, in the case of *P. aeruginosa* and *S. aureus,* complete killing occurred at higher MIC values. 

#### 2.3.2. Anti-Persister Activity of UA-CS NPs Towards the Biofilm-Associated Persister Cells

**Figure 7:**
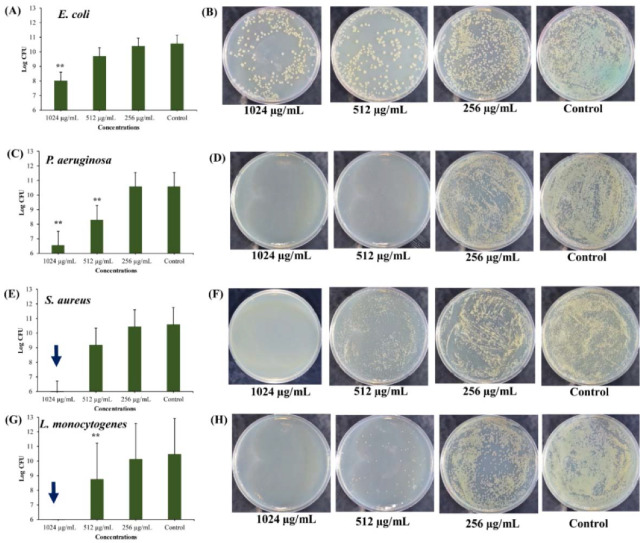
Killing of persister cells obtained from 5 days mature biofilm by different concentrations of UA-CS NPs. (**A**) Log CFU graph of *E. coli*, (**B**) *E. coli* colonies on TSA plate, (**C**) Log CFU graph of *P. aeruginosa*, (**D**) *P. aeruginosa* colonies on TSA plate, (**E**) Log CFU graph of *S. aureus*, (**F**) *S. aureus* colonies on TSA plate, (**G**) Log CFU graph of *L. monocytogenes*, and (**H**) *L. monocytogenes* colonies on TSA plate. After incubation, the UA-CS NPs treated cell culture were diluted up to 10^-6^ dilution and 100 µL mixture spread was plated on the TSA plate. Each plate shows a representative image of bacterial colonies. The arrow in the graphs indicate the complete killing of persister cells. Each bar is represented as the means of three replicates ± standard deviation. ** *p* < 0.01

Biofilm formation by most pathogenic bacteria results in the survival of the cells under adverse environmental conditions as well as counter effects the host immunological responses. Several strategies have been developed to inhibit biofilm formation as well as to eradicate established mature biofilms [20,60,61]. However, several reports have shown a failure in the treatment of biofilm-associated infections of pathogenic bacteria [62]. The main explanation for the lack of success of the antimicrobial therapy is the presence of persister cells in the biofilms as well as the presence of EPS, which hinders the penetration of the antimicrobial agent [62]. The killing of persister cells derived from the mature biofilm was UA-CS NPs concentration-dependent (Figure 7). Although there was no complete killing of the persister *E. coli* cells, a concentration-dependent inhibition in the log colony-forming unit (CFU) was observed (Figure 7A). In the presence of 1024 µg/mL of UA-CS NPs, the 2-log CFU of *E. coli* cells was reduced as compared to the control (Figure 7A). Similar results have been found for *P. aeruginosa*; although a concentration-dependent decrease in the log CFU occurred, a 4-log CFU reduction was observed at 1024 µg/mL of UA-CS NPs (Figure 7C). The complete killing of the persister cells of *S. aureus* and *L. monocytogenes* was at 1024 µg/mL UA-CS NPs (Figure 7E,G). We concluded that there was a reduction in the log CFU in the case of *E. coli* and *P. aeruginosa* at the MIC value of UA-CS NPs, whereas in the case of *S. aureus* and *L. monocytogenes*, complete killing occurred above the MIC value. The representative agar plate with bacterial colonies obtained from the cell culture treated with different concentrations of UA-CS NPs is shown in Figure 7B,D,F,H. 

#### 2.3.3. Anti-Persister Activity of UA-CS NPs towards Antibiotic-Induced Cells

**Figure 8:**
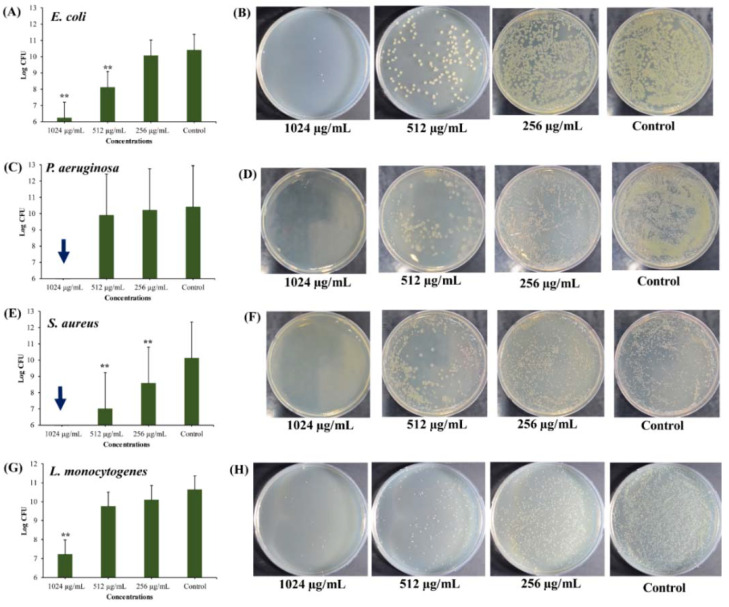
Killing of antibiotic-induced persister cells by different concentrations of UA-CS NPs. (**A**) Log CFU graph of *E. coli*, (**B**) *E. coli* colonies on TSA plate, (**C**) Log CFU graph of *P. aeruginosa*, (**D**) *P. aeruginosa* colonies on TSA plate, (**E**) Log CFU graph of *S. aureus*, (**F**) *S. aureus* colonies on TSA plate, (**G**) Log CFU graph of *L. monocytogenes*, and (**H**) *L. monocytogenes* colonies on TSA plate. After incubation, the UA-CS NPs treated cell culture were diluted up to 10^-6^ dilution and 100 µL mixture spread was plated on the TSA plate. Each plate shows a representative image of bacterial colonies. The arrow in the graphs indicate the complete killing of persister cells. Each bar is represented as the means of three replicates ± standard deviation. **, *p* < 0.01

Antibiotic treatment also leads to the formation of persister cells [63,64,65,66]. There are several mechanisms involved in the formation of persister cells upon exposure to chemicals or antibiotics, as explained in detail in the aforementioned references. The killing effect of UA-CS NPs on persister cells obtained from the antibiotic treatment was concentration-dependent in *E. coli*, *S. aureus,* and *L. monocytogenes* (Figure 8). Although no complete killing of *E. coli* persister cells occurred at 1024 µg/mL and 512 µg/mL of UA-CS NPs, a significant killing was observed at these concentrations with a reduction of 4- and 2-log of the cells (Figure 8A). The complete killing of *P. aeruginosa* persister cells occurred at 1024 µg/mL, whereas no significant reduction in the cells was observed below this concentration (Figure 8C). In the case of *S. aureus,* complete killing of persister cells occurred at 1024 µg/mL (Figure 8E). Almost 3-log and 2-log CFU of *S. aureus* cells were reduced at 512 and 256 µg/mL of UA-CS NPs, respectively (Figure 8E). The persister cells of *L. monocytogenes* at 1024 µg/mL were reduced by 3-log CFU (Figure 8G). The representative agar plate with bacterial colonies obtained from the cell culture treated with different concentrations of UA-CS NPs is shown in Figure 8B,D,F,H. The persister cells of *E. coli* produced by rifampin and tetracycline are tolerant upon treatment with the alpha-bromocinnamaldehyde, although it shows anti-persister activity against cells in the stationary phase of a batch culture and biofilms [67]. Although several naturally derived compounds are known to eradicate persister cells of different pathogenic bacteria, as summarized by Defraine et al. [4], very few marine-derived compounds are available as anti-persister agents. Rodrigues Felix et al. [68] screened 4400 marine products against replicating and non-replicating *Mycobacterium tuberculosis*, among which puupehenone-like molecule isolated from *Petrosia* exhibits anti-persister activity.

It was concluded that UA-CS NPs exhibited potential killing activity against different types of persister cells, either produced in naturally growing cultures or induced upon exposure to the antimicrobial agents. It was concluded that the killing of persister cells by UA-CS NPs occurred differently in the case of cells from stationary phase from batch cultures compared to the persister cells isolated from biofilms or induced by the antibiotic. The cells from stationary phase cultures of all bacteria were completely killed by the MIC (*E. coli*), above MIC (*P. aeruginosa*), and both MIC and sub-MIC (*S. aureus* and *L. monocytogenes*) of UA-CS NPs. However, variable killing occurred in persister cells obtained from biofilms or induced by the antibiotic. In addition, the colony morphology of *E. coli* observed on the tryptic soy agar (TSA) plate differed, with larger cells in the stationary phase as compared to the size of biofilm- and antibiotic-induced cells. Similarly, the colonies of *P. aeruginosa*, *L. monocytogenes,* and *S. aureus* were found to be slightly smaller in the case of biofilm- and antibiotic-induced cells. A possible explanation for the smaller colonies and tolerant properties of the cells towards the drug is the exposure to stressful environmental conditions such as biofilms and antibiotics [8,69,70]. In the case of the exposure to the environment or antibiotics of the biofilm and the stationary phase cells, the formation of persister cells is increased [69,70]. Although, the mechanism of the persister killing properties of UA-CS NPs against gram-positive and gram-negative pathogens is not known. However, previous experimental results showed that UA exhibits antibacterial activity towards actively growing planktonic cells by inhibiting the synthesis of RNA and DNA as well as by the disruption of the cytoplasmic membrane [14,52,71]. Furthermore, studies have shown that several compounds also kill persister cells directly by damaging or depolarizing the cytoplasmic membrane, affecting DNA-cross linking, generating cellular reactive oxygen species, or inactivating cellular enzymes [4,72,73,74,75]. Future studies are required to explore the killing mechanism of UA-CS NPs towards different types of persister cells of gram-positive and gram-negative pathogenic bacteria.

### 2.4. Effect of UA-CS NPs on Cell Morphology

**Figure 9:**
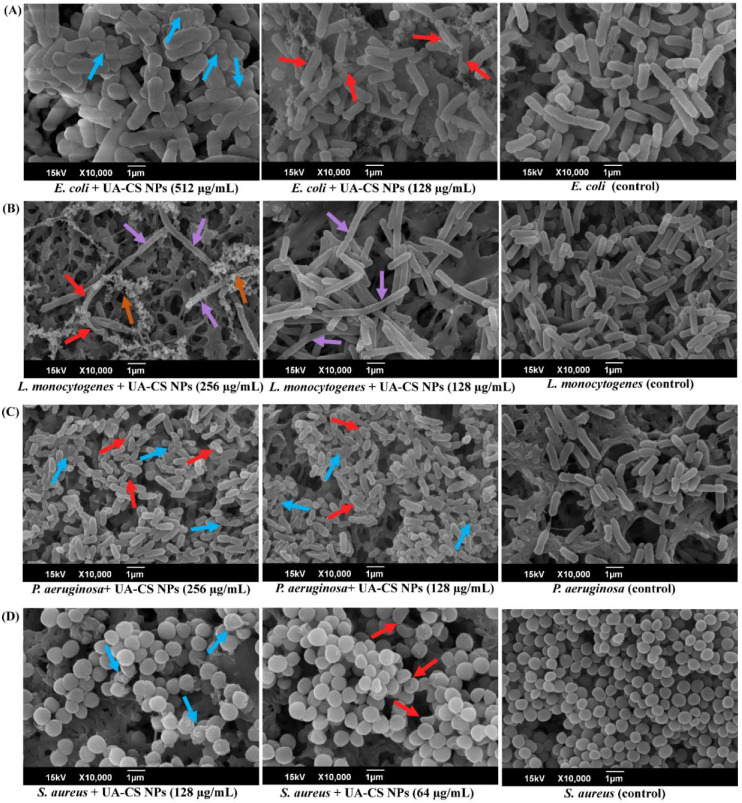
Microscopic analysis of cell morphology treated with sub-inhibitory concentrations of UA-CS NPs. Arrow indicated the change in cell morphology. (**A**) Cell morphology of the *E. coli* cell in the presence of sub-MIC UA-CS NPs, (**B**) cell morphology of the *L. monocytogenes* cell in the presence of sub-MIC UA-CS NPs, (**C**) cell morphology of the *P. aeruginosa* cell in the presence of sub-MIC UA-CS NPs, and (**D**) cell morphology of the *S. aureus* cell in the presence of sub-MIC UA-CS NPs. Deep-sky blue arrows indicate the dead cells, red color arrow-folding of the cell membrane, pink color arrow-elongated/filamented cells, dark orange color arrow-group of microcolonies.

The effect of UA-CS NPs on the morphology of gram-positive and gram-negative bacteria was examined using SEM. The sub-inhibitory concentration of UA-CS NPs for each bacterium was used to evaluate the changes in cell morphology (Figure 9). In the case of *E. coli*, at ½-MIC, most of the damaged cells were aggregated along with an increase in the thickness and diameter of the cells (Figure 9A). Furthermore, at ¼-MIC of UA-CS NPs, most of the *E. coli* cells were shrunken with irregular folds. Drastic morphological changes of *L. monocytogenes* cells occurred upon treatment with sub-MIC of UA-CS NPs. The results showed that at ½- and ¼-MIC, some *L. monocytogenes* cells were highly elongated and filamented as compared to the cells from the untreated control (Figure 9B). The shrinkage of the elongated *L. monocytogenes* cells at ½-MIC was also observed. Instead of elongation, numerous bead-like microcolonies of *L. monocytogenes* were surrounding the elongated cells upon exposure to ½MIC of UA-CS NPs (Figure 9B). In the case of *P. aeruginosa*, along with displaying lethality, some of the cells became irregular and shrunken in shape at ½- and ¼-MIC of UA-CS NPs as compared to the control cells (Figure 9C). Similarly, *S. aureus* cell morphology changed to irregular, oval, highly thickened, and swollen upon treatment with UA-CS NPs at ½- and ¼-MIC (Figure 9D). From the present study, we concluded that at sub-inhibitory concentrations, UA-CS NPs changed the cell morphology in different ways, such as producing elongation or filamentation (in the case of *L. monocytogenes*), irregular, swollen, thickening, and oval cells (in the case of *S. aureus*), and shrinkage of the cells (in the case of *E. coli* and *P. aeruginosa*).

Furthermore, unique morphological changes such as a shrunken surface with irregular folds, swollen, cell wall thickening, oval, and irregular shape have been reported for methicillin-resistant *S. aureus* upon exposure to UA [52]. The elongation and filamentation of *L. monocytogenes* is a unique finding in the present study. Numerous studies have reported the elongation and filamentation of *L. monocytogenes* cells in the presence of several environmental stress conditions such as temperature, salt, pH, and antimicrobials [76,77,78]. The elongation of *L. monocytogenes* cells by UA-CS NPs in the present study might be due to the inhibition of cell division. The genome of *L. monocytogenes* contains the *lmo*1071 gene that encodes the FtSW protein, which is responsible for cell division; when the gene is mutated cells become elongated [79]. Hence, in the presence of UA-CS NPs, the elongation of cells might be due to the inhibition of cell division. Another possibility to explain cell elongation or filamentation is that UA-CS NPs might be involved in the activation of the SOS response factor (e.g., YneA), which is known to be involved in cell elongation [80]. This morphological change in *L. monocytogenes* is an adaptive mechanism to combat and survive under stressful environmental conditions. Similarly, the thickening of the cell wall is another defensive mechanism against environmental stresses and antimicrobial agents, as it has been found in staphylococci in the presence of UA and some of the conventional antibiotics [52,81].

## 3. Conclusions

With the aim of improving the bactericidal activity of UA, its encapsulation into CS NPs was successfully conducted by a simple ionotropic gelification method. The encapsulation of UA into the CS NPs was confirmed by release assay, FTIR, XRD, and FE-TEM. The percentages of EE and LC were 24% and 5.2%, respectively. The shape of the UA-CS NPs was spherical and rough surface with an average diameter of 311.5 ± 49.9 nm. The UA-loaded CS NPs exhibited bactericidal activity towards gram-positive and gram-negative bacteria. Different types of persisters such as stationary phase cells, persisters obtained from mature biofilms, and antibiotic-induced *E. coli*, *P. aeruginosa*, *S. aureus*, and *L. monocytogenes* cells were killed by the MIC and sub-MIC of UA-CS NPs. The bacterial morphology was changed upon treatment with sub-MIC of UA-CS NPs, resulting in elongation (*L. monocytogenes*), irregular, swollen, thickening, and oval (*S. aureus*), or shrunken cells (*E. coli* and *P. aeruginosa*). Future studies are required to investigate the killing mechanism of UA-CS NPs towards different types of persister cells. Although, chitosan is a good biocompatible material, however, further studies are required to assess the cytocompatibility of UA-CS NPs in vivo and also its efficacy using some eukaryotic model organism. Based on the present findings, the encapsulated UA in CS NPs may be applied to treat the infection caused by biofilm-forming pathogenic bacteria. This study also provides a new direction for the loading of the least stable and hydrophobic drug into biocompatible chitosan for antimicrobial therapy.

## 4. Materials and Methods

### 4.1. Bacterial Strains, Growth Conditions, and Reagents

*Escherichia coli* (KCTC1682), *Listeria monocytogenes* (KCTC3569), *Pseudomonas aeruginosa* PAO1 (KCTC1637), and *Staphylococcus aureus* (KCTC 1916) were obtained from the Korean Collection for Type Cultures (KCTC, Daejeon, Korea). The growth media for the cultivation of *L. monocytogenes*, *S. aureus* and *P. aeruginosa* was tryptic soy broth (TSB; Difco Laboratory Inc., Detroit, MI, USA), whereas, for *E. coli*, Luria Bertani (LB) broth was used. Usnic acid, Tween 60, low molecular weight chitosan with 75–85% degree of deacetylation (CAS # 9012-76-4), and tripolyphosphate (TPP) were purchased from Sigma-Aldrich Co. (St. Louis, MO, USA). 

### 4.2. Preparation of Usnic Acid-Chitosan Nanoparticles (UA-CS NPs)

The methodology used for the preparation of UA-CS NPs was a single-step ionotropic gelification method as described earlier with slight modifications [22,39]. Briefly, low molecular weight chitosan (1.2% w/v) was initially dissolved in 1% acetic acid in a total volume of 100 mL of deionized water. The chitosan solution was continuously agitated at the room temperature for 12 h and added with 0.3 g Tween 60 to obtain a homogenous mixture. The mixture was stirred for 60 min at 50 ℃. The usnic acid (0.5%, w/v) solution prepared in dimethyl sulfoxide was slowly added and allowed to mix by stirring for 2 h at room temperature. Two different concentrations of TPP, which are 0.5% and 1.0%, were used for the preparation of UA-CS NPs. The solution of TPP was prepared in a total of 40 mL deionized water and was slowly added dropwise with continuous stirring for 2 h. The light yellow color mixture was centrifuged (13,000 rpm for 15 min) at 4 ℃. Similarly, CS NPs (unloaded with UA) was also prepared using 0.5% and 1.0% TPP. The obtained pellet was washed repeatedly for three times with deionized water and kept at −70 ℃ in a refrigerator to solidify. Finally, the frozen samples were freeze-dried using a freeze dryer (FD8518, ilShinBiobase Co. Ltd., Dongducheon, Korea).

### 4.3. Characterization of UA-CS NPs 

The detailed characterization of the newly prepared UA-CS NPs was carried out using different instrumentations and the procedures as described earlier [82,83]. The UV-visible-absorption spectroscopy was carried out by scanning over wavelength ranging from 200 to 800 nm using a microtiter plate reader (BioTek, Winooski, VT, USA). The morphology of UA-CS NPs was determined using field emission transmission electron microscopy (FETEM; JEM-2100F, JEOL, Japan). The particle size of CS NPs (loaded or unloaded with UA) and size distribution were determined by dynamic light scattering (DLS) using electrophoretic light scattering spectrophotometer (ELS-8000, OTSUKA Electronics Co., Ltd., Osaka, Japan). Similarly, the zeta potential of UA-CS NPs was measured using the electrophoretic light scattering spectrophotometer (ELS-8000; Otsuka Electronics Co. Ltd., Japan). Finally, the Fourier transform infrared spectrometer (FTIR, JASCO (FT-4100), Tokyo, Japan) analysis of UA-CS NPs was also performed at different frequencies ranging from 400 to 4000 cm^−1^ using 16 scans at a resolution of 4 cm^−1^. The X-ray diffraction patterns of the prepared CS NPs (loaded or unloaded with UA) were carried out using an X’Pert-MPD PW 3050 diffractometer (Phillips, Almelo, The Netherlands).

### 4.4. Determination of Encapsulation Efficiency and Loading Capacity

The amount of UA encapsulated in the CS NPs was determined by measuring the absorption spectrum using UV-vis absorption spectroscopy. The percentage of encapsulation efficiency (EE) and loading capacity (LC) were determined according to the previously explained procedure [22,48]. In the aqueous solution of hydrochloric acid (1M, 5 mL), UA-CS NPs (10 mg/mL) was dissolved and the mixture was boiled for 30 min at 95 ℃. After boiling, the samples were allowed to cool down followed by adding ethanol (1 mL) in the solution. The resultant mixture was pelleted down by centrifugation (9000 rpm, 30 min) at 25 ℃. The content of UA present in the supernatant was quantified by measuring the absorbance at 337 nm as described earlier [19]. The standard curve of UA was made by dissolving in phosphate-buffered saline (PBS, pH 7.4) and calibrated by measuring the absorbance at 337 nm. The percentage of EE and LC were calculated according to the following given equation;

(1) $$\mathrm{Percentage}\;\mathrm{of}\;\mathrm{EE}=\frac{\mathrm{Total}\;\mathrm{amount}\;\mathrm{of}\;\mathrm{UA}\;\mathrm{in}\;\mathrm{UA}-\mathrm{CS}\;\mathrm{NPs}}{\mathrm{Initial}\;\mathrm{amount}\;\mathrm{of}\;\mathrm{UA}}\times 100$$

(2) $$\mathrm{Percentage}\;\mathrm{of}\;\mathrm{LC}=\frac{\mathrm{Total}\;\mathrm{amount}\;\mathrm{of}\;\mathrm{UA}\;\mathrm{in}\;\mathrm{UA}-\mathrm{CS}\;\mathrm{NPs}}{\mathrm{Weight}\;\mathrm{of}\;\mathrm{UA}-\mathrm{CS}\;\mathrm{NPs}}\times 100$$

The experiment was conducted in triplicates and repeated three times

### 4.5. In Vitro Release of Usnic Acid from UA-CS NPs

The release kinetic of UA from the CS NPs was also determined by measuring the absorption at 337 nm as described earlier [19,22,48]. Furthermore, the release of UA was determined in PBS at three different pH values such as slightly alkaline (pH 7.4) similar to intestinal fluid, slightly acidic (pH 6.8) similar to colonic fluid, and acidic (pH 1.2) similar to the gastric fluid as explained earlier [34]. In brief, the powder of UA-CS NPs (10 mg) was placed in a tube containing 5 mL PBS (pH 1.2, 6.8 and 7.4) and incubated at 37 ℃ under gentle shaking. The total mixture in the tube was centrifuged (9000 rpm for 10 min) at every 2 h time interval and the absorbance of the supernatant was measured at 337 nm using microtiter plate reader. The UA release experiment was carried out in triplicates and repeated three times.

### 4.6. Minimum Inhibitory Concentration (MIC) Determination of UA-CS NPs

The bactericidal properties of UA-CS NPs were determined by microbroth dilution method as described earlier [82]. Briefly, the seed culture of each strain obtained from growing overnight in their respective growth media was being diluted in fresh culture media. These cultures were placed in the 96-well microtiter plate along with different concentrations of UA-CS NPs (ranging from 32 and 1024 µg/mL). The 96-well microtiter plate containing cells and UA-CS NPs were incubated under shaking (567 cycles per min: cpm) at 37 ℃ for 24 h in a microplate reader. After finishing the incubation, the optical density (OD) of the grown cell culture was measured at 600 nm in a microplate reader. The obtained OD value was analyzed to determine the MIC. Experiments were carried out in triplicate with two independent bacterial cultures and repeated three times.

### 4.7. Measurement of Persister Cells Formation

The effect of UA-CS NPs on the formation of persister cells from selected bacteria, which were induced by antibiotics such as ampicillin for *E. coli*, tetracycline for *P. aeruginosa* and *S. aureus*, and ciprofloxacin for *L. monocytogenes* were carried out according to the previously described procedure [67,70]. Briefly, the diluted (1:1000) overnight grown cell culture was grown at 37 ℃ to obtain OD_600_ ~1.0. These cells were treated with the MIC value of UA-CS NPs and different antibiotics at the concentration of 100 µg/mL and incubated at 37 ℃ for 3 h under shaking (250 rpm). The viability of each bacterial cells was enumerated by serial dilution (10^−6^ dilution) and spread plating (100 µL) on TSA plate. Similarly, the formation of persister cells from mature biofilm (grown for 5 days) was also measured as described below. The free-floating planktonic cells (obtained from the 5-day mature biofilm) from the 96-well microtiter plate were removed and the adhered cells in the wells were washed twice with fresh culture media and scraped off. These cell suspensions were serially diluted (10^−6^ dilution) in fresh culture media and spread plated (100 µL) on TSA plate. These TSA plates were incubated at 37 ℃ and the CFU of bacterial colonies that appeared on the TSA plate was determined. The experiment was conducted in triplicates and repeated three times.

### 4.8. Effect of UA-CS NPs on Stationary Phase Cells 

The isolation of stationary phase culture was carried out as described earlier with slight modification [67]. A single colony of each bacterial strain was inoculated into TSB or LB growth media (30 mL) and incubated under shaking conditions (200 rpm) at 37 ℃ overnight (16 h). The overnight grown cell cultures were diluted (1:1000) into their respective growth media and further incubated at 37 ℃ under shaking (200 rpm) until the cell culture reached the stationary growth phase (OD_600_ = 3.0). After incubation, the full-grown cells were harvested by centrifugation (13000 rpm for 10 min). The obtained cell pellets were washed three times with PBS (pH 7.4). Finally, the cell pellets were re-suspended in PBS and adjusted the OD to 0.2. These cells (240 µL) were placed in a 96-well microtiter plate along with different concentrations of UA-CS NPs (ranging from 256 and 1024 µg/mL) and incubated under shaking (567 cpm) at 37 ℃ for 6 h in a microplate reader. After incubation, the cells were serially diluted (up to 10^−6^ dilution) in fresh TSB and spread plated (100 µL) on the TSA plate. These plates were incubated overnight and appeared colonies were counted. The experiment was conducted in triplicates and repeated three times.

### 4.9. Eradication of Biofilm-Associated Persister Cells by UA-CS NPs

Firstly, the mature biofilm of *P. aeruginosa*, *S. aureus*, *L. monocytogenes,* and *E. coli* was allowed to establish in their respective growth media at 37 ℃ for 5 days as described earlier [83]. The free-floating planktonic cells from the fully formed mature biofilm were removed carefully. The remaining surface-attached biofilm cells were washed two times with fresh growth media and the cells were scraped off. The cell suspension in TSB was centrifuged (13000 rpm for 10 min) and washed three times with PBS. The obtained pellets were re-suspended in PBS and adjusted the OD to 0.2. These cells (240 µL) were placed in the 96-well microtiter plate along with different concentrations of UA-CS NPs (ranging from 256 and 1024 µg/mL) and incubated under shaking conditions (567 cpm) at 37 ℃ for 6 h in a microplate reader. After incubation, the cells were serially diluted (up to 10^−6^) in fresh TSB and spread plated (100 µL) on the TSA plate. These plates were incubated overnight and counted the appeared colonies. The experiment was conducted in triplicates and repeated three times.

### 4.10. Eradication of Antibiotic-Induced Persister Cells by UA-CS NPs

The antibiotic-induced persister cells were treated with UA-CS NPs and the effect on its number was enumerated by counting the cells on the TSA plate. The procedure was executed as described earlier with slight modifications [70]. The seed culture of each bacterial strain obtained after being grown overnight in growth media was diluted up to 1:1000 into their respective growth media and further incubated at 37 ℃ until the cell culture reached the stationary growth phase (OD_600_ = 3.0). Previous reports showed that when stationary phase cell culture was treated with antibiotics, additional persister cell formation was formed as a result [69,70]. These grown cell cultures were treated with different antibiotics such as tetracycline for *P. aeruginosa* and *S. aureus* at the working concentrations of 100 µg/mL, whereas the cell culture of *L. monocytogenes* and *E. coli* were treated with ciprofloxacin and ampicillin at 100 µg/mL. The antibiotic-treated cell cultures were grown under shaking conditions (250 rpm) at 37 ℃ for 3 h. The antibiotic-induced persister cells were harvested by centrifugation (13000 rpm for 10 min) followed by washing with PBS three times. Obtained pellets were re-suspended in PBS and adjusted the final OD with a value of 0.2. These antibiotic-induced persister cells were treated with different concentrations (ranging from 256 and 1024 µg/mL) of UA-CS NPs and allowed to grow at 37 ℃ for 6 h. After incubation, the cell culture was serially diluted up to 10^-6^ dilution and spread plated (100 µL) on a TSA plate. The TSA was plate incubated overnight at 37 ℃ and their CFU were determined. The experiment was conducted in triplicates and repeated three times.

### 4.11. Analysis of Cell Morphology of Bacterial Cells Treated by UA-CS NPs 

The impacts of UA-CS NPs on the cell morphology of bacteria was checked under scanning electron microscopy (SEM) as described earlier with slight modification [84]. Briefly, the overnight grown cells were diluted (1:100) in their respective growth media and treated with sub-MIC of UA-CS NPs. These cell cultures were incubated at 37 ℃ for 4 h under shaking conditions (200 rpm). UA-CS NPs treated cell culture (1 mL) were placed in the 24-well plate containing nylon membrane (0.5 × 0.5 cm) and incubated for 5 h at 37 ℃. The bacterial cells on the nylon membrane surface were fixed using formaldehyde (2%) and glutaraldehyde (2.5%) at 4 ℃ overnight. The cells had been fixed using the above-fixing agents were washed thrice using PBS; these fixed cells were then further dehydrated using increasing concentrations of ethyl alcohol. These nylon membranes were freeze-dried using a freeze dryer (FD8518, ilShinBiobase Co. Ltd., Dongducheon, Korea). These membranes were directly affixed to SEM stubs and coated with gold for 120s using ion-sputter (E-1010, Hitachi, Japan). Finally, bacterial cell morphologies were visualized by using JSM-6490LV (JEOL, Tokyo, Japan) at the magnification of × 5000 and voltage of 15 kV.

### 4.12. Statistical Analysis

The GraphPad Prism 7.0 (GraphPad Software Inc., San Diego, CA, USA) was used to plot all graphs. Furthermore, the statistical analysis of each experimental data was also executed using one-way ANOVA, and results were presented as means ± SD. ** *p* < 0.01 and * *p* < 0.05 were considered as significant. 

### 4.13. Abbreviations

UA, usnic acid; CS, chitosan, NPs, nanoparticles; CS NPs, chitosan nanoparticles; UA-CS NPs, usnic acid-chitosan nanoparticles; SEM, scanning electron microscopy; EE, encapsulation efficiency; LC, loading capacity; FETEM, field emission transmission electron microscopy; FTIR, Fourier transform infrared spectrometer; DLS, dynamic light scattering; MIC, minimum inhibitory concentrations.
